# Aligning complex processes and electronic health record templates: a quality improvement intervention on inpatient interdisciplinary rounds

**DOI:** 10.1186/s12913-015-0932-y

**Published:** 2015-07-13

**Authors:** Hilary J. Mosher, Daniel T. Lose, Russell Leslie, Priyadarshini Pennathur, Peter J. Kaboli

**Affiliations:** Comprehensive Access and Delivery Research and Evaluation (CADRE) Center at the Iowa City VA Healthcare System and VA Quality Scholars Fellowship Program, Iowa City, IA USA; Department of Internal Medicine, University of Iowa Carver College of Medicine, Iowa City, IA USA; College of Nursing, University of Iowa, Iowa City, IA USA; Department of Industrial Engineering, University of Iowa College of Engineering, Iowa City, IA USA; Iowa City VA Healthcare System, 601 Highway 6 West, Mailstop 152, 52246-2208 Iowa City, IA USA

**Keywords:** Interdisciplinary rounds, Medical education, Quality improvement, Electronic health records

## Abstract

**Background:**

Interdisciplinary rounds (IDR) with documentation have become a standard of care, but the process has been incompletely described in academic general medical settings. Checklists are promoted, yet standardized formats may not reflect the variability and work flow of rounds or support the cognitive development of medical trainees. We describe IDR processes in an academic general medicine inpatient setting and present a rapid cycle quality improvement (QI) project that improved IDR documentation rates in the electronic health record.

**Methods:**

The project team observed existing daily IDR rounds on two medical inpatient units at the Iowa City VA Medical Center, with three resident teams and maximum census of 42 patients. The major intervention was a redesigned note template, with accompanying resident educational materials. The primary outcome was note completion rates by charge nurses; IDR team member satisfaction and participation, discussion time and balancing metrics (i.e., excess bed days of care, length of stay, and 30-day readmissions) were also assessed.

**Results:**

An electronic template and accompanying educational materials designed to parallel the heuristic problem-solving activities of the IDR team led to improvements in IDR note completion rates from 27 to 69 %. Team member satisfaction was high and participation was stable. Discussion time per patient increased modestly, but varied widely between resident teams and by patient. Balancing metrics were unchanged. Unstructured evaluation indicated that documentation times were reduced, and IDR documentation became more timely and useful.

**Conclusions:**

IDR notes designed to support the problem-solving processes of an interdisciplinary group improved the timeliness and perceived value of IDR documentation and met regulatory standards. Aligning complex processes and educational and documentation needs during IDR may create an efficient opportunity for sustainable interdisciplinary work and learning in an academic setting.

**Electronic supplementary material:**

The online version of this article (doi:10.1186/s12913-015-0932-y) contains supplementary material, which is available to authorized users.

## Background and significance

Interdisciplinary rounds (IDR), [1] also referred to as “interdisciplinary team meetings”, [2] “multidisciplinary rounds”, [3] and “discharge rounds” [4] are standard of care in the inpatient setting. Interdisciplinary care is a “Provision of Care, Treatment, and Services” standard with a measure of success requirement of The Joint Commission (TJC), specifying that indisciplinary care be documented [5]. The general objective of IDR in the inpatient setting is to bring together disciplines responsible for the care of hospitalized patients, with the aim of improved communication during the inpatient stay and better care transitions within and outside the hospital; how IDR are best conducted or documented is not known.

A survey of studies on IDR reveals a wide range of settings, processes, and approaches to implementing, improving, and evaluating IDR. A systematic review on information technology (IT) tools demonstrates the heterogeneity of purpose, settings, and measures related to IDR [6]. In the intensive care unit (ICU), IDR tend to focus on communication between physicians, bedside nurses, and additional staff (e.g. respiratory therapy) regarding medical management and advancing care [7, 8]. In non-ICU settings, some IDR models have had limited physician participation and been led by Clinical Nurse Specialists [9].

Several studies have described structured rounds, led by clinical champions, which employ checklist formats to guide interdisciplinary communication in general [10, 11] or aimed at achieving specific outcomes [3, 12]. Checklists present an attractive format for designing templated documentation. However, questions remain about the appropriateness of checklists to guide interdisciplinary work. Excess structure can lead to a loss in cognitive focus in that participants omit highly relevant information if it is not part of the structured or check-listed format [2, 12]. Better understanding of the processes and contexts of IDR and applicability of checklists is central to explaining if and how these improve collaborative communication [13].

In addition to being important to patient care quality, safety, and efficiency, interdisciplinary work is a recognized professional competency [14]. How practitioners learn and develop skills in interdisciplinary work is not well-described; we consider it likely that various IDR models have differential effects on skill development. Participant outcomes reported in the literature include situational awareness [8], satisfaction [15], and knowledge of core measures [3]. The educational and training value of IDR may be important to consider as institutions make decisions to employ dedicated care navigators or discharge planners to supplement or supplant interprofessional teams in coordinating care.

Local implementation or improvement of IDR is challenging in part due to the heterogeneity of structures, processes, and outcomes of IDR as described in the literature. While the IDR process may facilitate quality improvement (QI) targets (e.g. core measure compliance [3], decreased length of stay [16], or timely discharge [17]) in a given setting, translating these successes to other settings requires understanding the local environment and potential unanticipated effects, such as time burden, staff perceptions, and educational outcomes. Few published descriptions of IDR report balancing measures when describing improvements in targeted outcomes.

In 2013, our QI team was tasked with improving the IDR process after a tracer audit found the hospital did not meet TJC standards for interdisciplinary care planning documentation; specifically, documentation was inconsistently completed. This opportunity allowed us to observe IDR to identify key tasks and content that should be documented, and to explore barriers and facilitators of documentation. In our local setting, IDR have been performed for years and functioned without defined structure, leadership, or oversight. We hypothesized that through close observation of the IDR process, we could identify means of standardizing and improving IDR documentation rates without negative effects on the process, perceived value, or standard quality metrics. The primary outcome targeted was documentation rate. We describe our IDR observations and our rapid cycle improvement experience to inform future work on implementation and assessment of IDR processes in general medical units across academic and community settings.

## Methods

### Project design

This QI project consisted of structured observations and measurement of interdisciplinary rounds (IDR) with iterative Plan-Do-Study-Act (PDSA) cycles of change to workspace, education and training materials, and IDR documentation process and format.

### Settings and participants

The project involved two medical inpatient units at the Iowa City Veterans Administration (VA) hospital, encompassing three resident teams with total maximum census of 42 patients. The interdisciplinary care team included resident physicians, charge nurses, nurse managers, social workers, and representatives from supporting services (i.e., palliative care; utilization review, pharmacy, home health, diabetes education, dietician services, and respiratory, physical and occupational therapies). IDR are held in a designated room between 11:15 am and 12:15 pm, after medical rounds are completed. Senior residents lead the discussions, with each resident team meeting sequentially with the IDR team. Prior to the intervention, residents were provided no formal instruction in how to lead rounds. Charge nurses were responsible for completing the interdisciplinary documentation, and typically performed this task following rounds, often at the end of the day. The QI team consisted of two physicians, a nurse, a human factors engineer, and a QI specialist with training in health administration.

### Patient population

The patient population served by the three inpatient medical teams was predominantly male (96 %) with a mean age of 65.5 years; the most common admitting diagnoses were heart failure, community acquired pneumonia, and chronic obstructive pulmonary disease. General medicine teams care for all patients, including ICU, cardiac, and oncology patients with appropriate consultation. The hospital serves a rural population with 65 % of patients being discharged to rural settings. Teams admit in sum 200-250 patients per month with an average hospital length of stay (LOS) of 3.7 days; 65 % are acute admissions and 35 % are observation status.

### Data sources

Data regarding day to day functioning of IDR were collected during observation using a structured data collection worksheet (Additional file 1) developed by the QI team. IDR participant perspectives were obtained by survey and unstructured interviews. Note completion counts were obtained from the Computerized Patient Record System (CPRS), the electronic health record used across Veterans Health Administration (VHA). Data on ward days of care (a proxy for patient census), excess bed days of care, hospital LOS, and 30-day readmissions were obtained from hospital reports as part of ongoing administrative and Quality Management efforts.

A survey was developed with five questions on team functioning using a five-point Likert scale with responses ranging from strongly disagree (1) to strongly agree (5) (Table 1). Three open ended questions were included in the pre-implementation survey; “*What information do you need from Interdisciplinary Rounds to help you do your job*?”, “*What potential problems are avoided in your line of work by coming to Interdisciplinary Rounds*?”, and “*Please list 1-2 ideas on how Interdisciplinary Rounds could be improved to meet your needs*.” Two open ended questions were included in the post-implementation survey; “*What behaviors have Residents shown in Interdisciplinary rounds that maximize the meetings effectiveness?* and “*Please List one or two ideas on how Interdisciplinary Rounds could be improved to meet your needs.”* Surveys were distributed to all non-physician members of the IDR team present on a single day pre (N = 9) and post-intervention (N = 11).

**Table 1:** Responses to interdisciplinary rounds survey by team members

	Pre-intervention	Post-intervention
	N = 9	N = 11
Team conversations are focused on providing high-quality care*	4.1	4.2
The Team effectively coordinates patient discharges	4.0	4.4
Team members feel comfortable raising questions, issues, or concerns	4.3	4.3
Team members have a clear understanding of *my* role	3.9	4.2
Team members volunteer valuable information (when relevant)	4.1	4.2

*For the following statements concerning IDRs, indicate how much you agree or disagree (1 = strongly disagree to 5 = strongly agree)

### Data elements and outcomes

The targeted outcome measure was the IDR note completion index, as the impetus of the project was to improve documentation rates of interdisciplinary care planning to meet TJC standards. The index was calculated as the number of IDR notes completed per day (Monday through Friday) divided by the ward days of care calculated for that day. Additional outcomes included IDR team member satisfaction and participation, discussion time, and standard quality metrics of length of stay, and 30-day readmission rate, and excess bed days of care. Participation and time were recorded during structured observations (Additional file 1). Participation rate was calculated as the number of distinct specialties speaking per patient discussed. Participation was scored as the first comment by a member discipline on a patient; additional comments by a member of the same discipline on the same patient were not scored; participation of the resident physician leading rounds was assumed and not scored. The participation ratio reflects the sum of discipline counts for each patient, divided by the total number of patient discussions that day. Discussion time was the total time in seconds elapsed during a single patient discussion. Excess bed days of care represent the proportion of hospital bed days during which patients did not meet criteria for acute care.

### Interventions

**Fig. 1:**
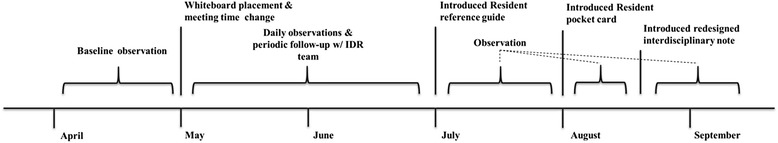
Interdisciplinary Rounds Project Design and Timeline

The project timeline included three PDSA cycles including structured observations of the IDR process (Fig. 1). The first PDSA cycle incorporated a new white board system, conceptualized by the physician leaders as a way to visually represent a shared mental model of moving patients from admission to discharge. The new boards were installed with the intent that the team would integrate them into the daily workflow. The second PDSA cycle involved development and refinement of resident education materials, including handouts and pocket cards outlining the resident role during IDR that followed the content of the EHR note template (Additional file 2). The education materials were based on observations of residents considered by the interdisciplinary care team to be highly effective, and thus were descriptive rather than prescriptive. The third PDSA cycle involved redesign of the IDR note template. This original template was a long, detailed, check-list that included items unrelated to topics discussed in IDR. The length, detail, and content precluded real-time completion of the note during IDR rounds. Based on structured observations of IDR as well as unstructured interviews with the IDR team, the QI team redesigned the template to include common issues and reflect the order of typical discussions, especially those led by residents felt by the IDR team to be most effective. The template was iteratively trialed and modified based on feedback from the charge nurses completing the note (Additional file 3). Resident education materials were designed to parallel the template. As part of continuous QI, resident education materials were expanded into a short video podcast to be shown at the beginning of each month-long rotation [18].

### Analyses

Pre- and post-intervention note completion rates were analyzed using statistical process control (SPC) methods, specifically p-charts [19]. IDR team satisfaction was surveyed on a Likert scale and mean scores summarized. Participation rates were presented as a simple linear regression of rates over time. Mean discussion time was compared using the t-test. Length of stay and hospital readmissions were analyzed as individuals charts, and excess bed days of care as a p-chart. The authors had full access to and take full responsibility for the integrity of the data. All analyses were conducted using SAS® statistical software version 9.2 (Cary, NC), with SPC charts created using SPC-XL Excel templates, an add-on by SigmaZone. The project was reviewed by the University of Iowa Institutional Review Board and approved by the Iowa City VA Healthcare System Research and Development Committee. The project and manuscript development were guided by the Standards for Quality Improvement Reporting Excellence (SQUIRE) [20].

## Results

### Intervention acceptance

In PDSA cycle one (month two), a white board was designed to represent flow through stages of care, with magnetic boards that could be moved across a larger white board representing the admission day, daily care, and discharge day. This design was not adopted by the team. Reasons for not adopting it included difficulty due to the crowded small room and accessing the board to physically move magnetic icons; no single team member taking ownership of the process; and each team-member’s unique methods of recording information on paper for his or her own daily tasks. Ultimately, the team did use the white board to list patients by the three resident teams with some adoption of magnetic icons to mark patient needs (e.g., transportation, diabetes education, physical therapy, oxygen).

In PDSA cycle two (month four), resident education materials were adopted, with residents observed actively using the pocket cards. In PDSA cycle three (month five), the IDR note template was fully implemented. Both the education materials and note template were integrated into observed dailywork flow (Fig. 1).

### Note completion rates

**Fig. 2:**
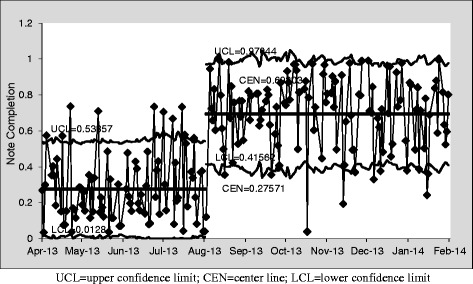
Interdisciplinary Round Notes Completed per Ward Days of Care (p-chart)

The pre-invention IDR note completion rate, as measured by number of patients with a note per ward days of care, was 27 % over an 85-day observation period; post-intervention completion rate was 69 % over 119 days, with the post-intervention center line exceeding the pre-intervention upper confidence limit. Completion rates were measured for the five months following introduction of the new template, during which time the higher completion rates were sustained, although with daily variability (Fig. 2).

### IDR team satisfaction

The survey results show a high level of satisfaction with rounds in all 5 themes (Table 1). Pre-intervention free-text responses (Additional file 4) indicated that residents should more clearly identify the anticipated date of discharge and discharge needs. Recommendation to improve rounds included better role-clarification, arriving on time to start IDR, and starting IDR earlier in the day. Avoiding last minute requests and referrals was seen as an important goal. Post-intervention comments (Additional file 5) were positive regarding the revised process, yet identified areas for continuous improvement (e.g., more efficient use of time, bigger room, and continued efforts to identify of patient needs earlier in the hospitalization).

### Participation during IDR

**Fig. 3:**
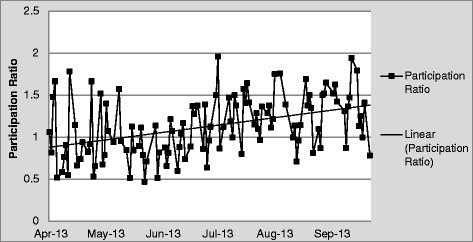
Participation During Interdisciplinary Rounds

Disciplines with the most participation were nursing, followed by social work and physical therapy. Considerably fewer comments were made by representatives from utilization review, palliative care, pharmacy, diabetes educator, respiratory therapy, nutrition, and occupational therapy (data not shown). Participation was stable during the project period (slope = 0.003, R^2^ = 0.172) (Fig. 3).

### Time spent during IDR

**Fig. 4:**
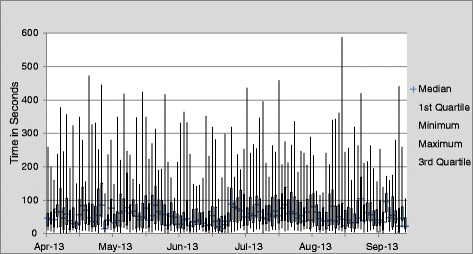
Time Variability of IDR Discussions (in seconds)

The mean time spent in discussion per patient increased slightly over the course of the project period (mean discussion time was 64 seconds per patient compared with 72 seconds per patient following the intervention (*p* = 0.0007) (Table 2). We observed high variability in time spent discussing each patient throughout the course of our observations. Time per patient varied by resident team, as well as by patient, ranging from 1 second to 9 minutes and 48 seconds. The box-and-whisker plot illustrates this time variability, with no decrease in variability over the course of our study (Fig. 4).

**Table 2:** Mean, median, and modal time in seconds per patient discussion pre- and post-intervention

Duration of individual patient discussion (in seconds)	Pre-intervention	Post-intervention
	N = 1581	N = 1424
Mean (SD)*	64.2 (64.5)	72.2 (64.8)
Median	44	53
Mode	15	17

*P-value for differences in means =0.0007

### Balancing measures

**Fig. 5:**
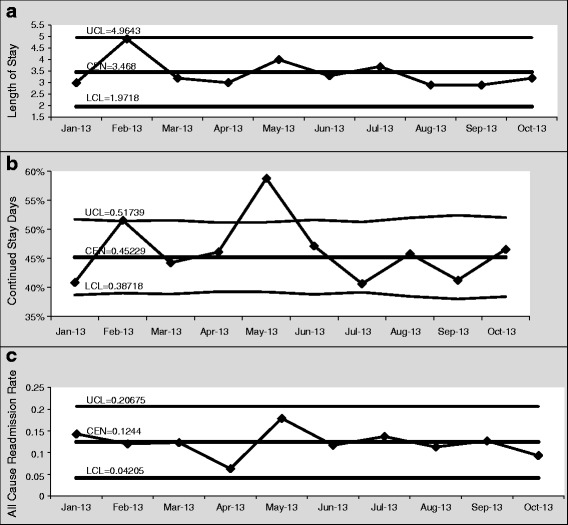
**a** Hospital Length of Stay (individuals chart). **b**: Excess Bed Days of Care Measured by Acute Continued Stay Reviews Not Meeting Criteria (p-chart). **c** 30 Day Hospital Readmission Rate (individuals chart)

Hospital length of stay, 30-day readmission rates, and excess bed days of care did not indicate any special cause variation that could be attributed to the IDR QI process (Fig. 5a-c).

### Qualitative and unanticipated results

Unstructured interviews revealed that charge nurses saw a decrease in time burden to complete notes of an estimated 30 to 60 minutes per day. Further, because notes were completed by early afternoon, bedside nurses began using these notes to obtain information for daily care and discharge plans. Free text responses to the post-intervention survey suggested positive trends in residents’ performance: “most have been providing appropriate information and listen to requests/recommendations;” “receptiveness to input from team;” “outlining key issues;” “open to questions;” “a collaborative perspective” (Additional file 5).

### Products and dissemination

At project completion, the note template, resident education material, and an implementation toolkit were made available in adaptable fomats to all VA facilities [18]. The QI team was invited to discuss the project with a national audience through the VHA Office of Systems Redesign and Improvement and has been contacted regularly regarding adopting the materials.

## Discussion

Our efforts were successful in improving and sustaining completion rates of interdisciplinary care documentation, as measured by IDR documentation index. We attribute this success to redesign of the document template such that it is easily completed in real-time or shortly following each patient discussion. Moreover, the template mirrored instructions to residents on how to lead rounds, which may have facilitated efficient note completion. That the secondary outcomes of IDR team member satisfaction and participation were stable indicated the intervention did not negatively disrupt IDR. Similarly, conventional quality measures of length of stay, excess bed days of care,and 30-day readmissions were unaffected by the intervention, consistent with prior studies that found these metrics insensitive to changes in interdisciplinary care processes [21].

The duration and time variability we observed in our IDR were similar to that documented by Sen *et al* in a trauma center, who showed a median of 13 seconds (range, 2 - 233 seconds) with 96 % of discussions lasting less than a minute [22]. The authors connected short duration and goal-focused communication with the sustainability and success of IDR.

The observation that perceived high-quality rounds were brief and highly-variable was foundational to our intervention, as it challenged our assumptions about the potential value of a more comprehensive document template or structured, one-size-fits all IDR format. At the project outset we anticipated designing a checklist and template to standardize patient discussions during IDR. However, our early unstructured discussions with IDR members suggested that perceived effectiveness was based more on having targeted, patient-centered discussions, and less on comprehensiveness or adherence to a standard list. Thus, we designed a template based on a heuristic, rather than algorithmic, checklist.

A heuristic checklist provides general instructions for how to structure information in order to allow a medical team to arrive at provisional action plans, but does not prescribe sequential or contingent steps, as algorithmic checklists do. Thus, heuristic checklists provide greater space for cognitive processing, whereas an algorithmic checklist is followed in stepwise fashion for each patient, by each practitioner, each time.

We propose that the work of a functioning IDR team is best characterized as a complex task achieved in “highly flexible and fluid ways” [12]. This work is supported by approaches that emphasize free text, both within documentation templates and metaphorically within the IDR process itself, allowing for adaptability and free exchange of information guided, not by an algorithmic checklist, but by the knowledge and experience of participating professionals [23]. Likewise, the relative value of heuristic or algorithmic approaches to achieve diverse goals in various settings bears further examination. The optimal amount of structure to facilitate comprehensive discussion when needed, but allow enough flexibility to permit efficient, very brief discussions, likely varies by setting.

The success of the intervention, which was adopted locally and is in the process of being adapted to other VA settings, may be attributable both to the reduced time burden of using this template, as well as to improved alignment of daily workflow and training goals, through a heuristic checklist that accurately reproduced shared communication patterns.

Shared communication patterns are foundational in medical education, as evidenced by formal training in the History and Physical (H&P) format as well as the Subjective, Objective, Assessment, and Plan (SOAP) format and its variants in daily verbal and written progress reports. The experience of structured rounds and use of scripts [10, 11, 24] suggests the potential of similarly codifying IDR communication.

In our resident education materials, we present the Identify, Summarize, Discuss, and Ask (ISDA) heuristic in IDR as analogous to a SOAP presentation on medical rounds [18]. Providing training and guidance to residents about how to communicate with an established IDR team and reinforcing this communication heuristic through a documentation template may contribute to development of interprofessional competence through “learn-as-you-go training”. Our observation that potentially valuable and functional rounds can be performed in a minimum of time and without use of an extensive script, in contrast to that described by O'Leary and Stein [10, 11], suggest that better understanding of the optimal design and content of IDR might be gained through comparative studies of the two approaches.

In addition to producing templates and materials that have been or are being adopted outside of our local setting, our work on developing documentation based on close observation of existing workflow informs broader efforts to establish, structure, assess, and improve IDR teams as part of interprofessional education [25] especially in academic centers in which physicians-in-training, and often attending physicians, constitute a transient workforce interacting with a more permanent interprofessional group.

Our study is limited by its lack of measures to assess team function. Although measures of teamwork are available, the QI team was not equipped to employ these measures and was unsure if they would be sensitive to the changes proposed. Bedside nurses were not included in the process due to the difficulty in coordinating rounding schedules. We did not have direct measures of the utility of the revised documentation; bedside nurses did spontaneously begin referring to the document to obtain information on the daily plan, suggesting increased utility. We did not directly assess resident attitudes or educational outcomes. In addition, no direct patient-related outcomes were targeted. Although balancing metrics were used to assure no negative consequences on efficiency, the intervention was not intended to improve any specific clinical outcomes or powered to do so. The limitations in our outcome measures reflect the broader challenge of effectively evaluating interprofessional care and education, articulated in a newly released IOM report [25]. Lastly, the project was completed at a single, academic-affiliated VA hospital which may not be generalizable to all other hospital settings.

## Conclusion

Our experience provides a real-life example of the challenges and opportunities with using documentation requirements and EHRs as a lever for improving care processes. Ultimately, the templates and education materials created were designed to be as brief and general as possible, to support heterogenous discussions while enabling efficient completion of required documentation; the utility of the completed document remains unclear. Our experience echoes that of Ash *et al*, who describe “loss of overview” and inability to accommodate the “fluid and contingency-driven nature of health care work” as potential harms of information technology [12]. Our finding that a well-functioning team performs care coordination discussions in highly variable ways depending both on personnel and patients suggests that interdisciplinary work follows a heuristic rather than algorithmic script. The improved rate and timeliness of documentation with the new note template indicate that a short template with increased opportunity for free text may be more efficient and effective for capturing high-level or collaborative work. Variability in fact may be a positive characteristic of complex team processes; efforts to improve these processes may need to balance standardization and flexibility in order to maximize team performance.

### Data availability

Original data are available to researchers with Veterans Administration clearance from Russell Leslie.

## Additional files

Additional file 1:
**Appendix A.** Data Collection Instrument for Observations. Additional file 2:
**Appendix B.** Resident Education Poster. Additional file 3:
**Appendix C.** Electronic health Record Interdisciplinary Rounds Note Template. Additional file 4:
**Appendix D.** Pre-Intervention Survey Free-Text Responses. Additional file 5:
**Appendix E.** Post-Intervention Survey Free-Text Responses.

## Abbreviations

ACGME: Accreditation Council for Graduate Medical Education
H&P: History and Physical
ICU: Intensive Care Unit
IDR: Interdisciplinary rounds
ISDA: Identify, Summarize, Discuss, and Ask
LOS: Length of stay
PDSA: Plan, Do, Study, Act
QI: Quality improvement
SQUIRE: Standards for Quality Improvement Reporting Excellence
SOAP: Subjective, Objective, Assessment, Plan
TJC: The Joint Commission
VA: Veterans Administration
