# Supplementary material for: Aligning complex processes and electronic health record templates: a quality improvement intervention on inpatient interdisciplinary rounds
Source: BMC Health Serv Res. 2015 Jul 13;15:265. doi: 10.1186/s12913-015-0932-y (PMC4499441; doi:10.1186/s12913-015-0932-y)
Supplement: Additional file 1: — Appendix A. Data Collection Instrument for Observations. [file 12913_2015_932_MOESM1_ESM.docx]

**Additional file 1: Data Collection Instrument for Observations**

Start Times Blue: Red: White: End Time: Date: Observer:

| **Patient** | **Diagnosis** | **Who spoke** | **Hospital Care** | | **Discharge Planning** | | | **Time &**  **Comments** |
| --- | --- | --- | --- | --- | --- | --- | --- | --- |
|  |  |  | **Daily Care Plan** | **Other** | **Transportation** | **Home Needs (Meds, O2, Safety) or: Placement** | **Expected DC date** |  |
| **Admit day**  **Daily**  **D/C day** | CHF | RN, SW, PT, Pharm, HC, UR, PC, NUT, OT, D-RN | Medication change  Procedure  Consult | Foley/Tele  Lines | Self,  DAV, Ambu  Other | PT, HC, Abx, O2  Placement: CLC, SNF  Other: | Today  + 2 days | 34 seconds |
| **Admit day**  **Daily**  **D/C day** |  | RN, SW, PT, Pharm, HC, UR, PC, NUT, OT, D-RN | Medication change  Procedure  Consult | Foley/Tele  Lines | Self,  DAV, Ambu  Other | PT, HC, Abx, O2, SN  Placement: CLC, SNF | Today  + ___days |  |
| **Admit day**  **Daily**  **D/C day** |  | RN, SW, PT, Pharm, HC, UR, PC, NUT, OT, D-RN | Medication change  Procedure  Consult | Foley/Tele  Lines | Self,  DAV, Ambu  Other | PT, HC, Abx, O2,SN  Placement: CLC, SNF | Today  + ___days |  |
| **Admit day**  **Daily**  **D/C day** |  | RN, SW, PT, Pharm, HC, UR, PC, NUT, OT, D-RN | Medication change  Procedure  Consult | Foley/Tele  Lines | Self,  DAV, Ambu  Other | PT, HC, Abx, O2,SN  Placement: CLC, SNF | Today  + ___days |  |
| **Admit day**  **Daily**  **D/C day** |  | RN, SW, PT, Pharm, HC, UR, PC, NUT, OT, D-RN | Medication change  Procedure  Consult | Foley/Tele  Lines | Self,  DAV, Ambu  Other | PT, HC, Abx, O2, SN  Placement: CLC, SNF | Today  + ___days |  |

RN=Registered Nurse, SW=social worker, PT=physical therapy, Pharm= Pharmacy, HC=home care, UR= Utilization Review, PC=palliative care, NUT= Nutritionist, OT=occupational therapy, D-RN=diabetes nurse Abx= Antibiotics, SN= Skilled Nursing Homecare, Self= Patient’s own transportation (e.g. family, friend), DAV=disabled American Veterans Van, Ambu= Ambulance, CLC=community living center, SNF=skilled nursing facility, XS days=excess days of care, ABX=antibiotics

General Notes:
