# Supplementary material for: Aligning complex processes and electronic health record templates: a quality improvement intervention on inpatient interdisciplinary rounds
Source: BMC Health Serv Res. 2015 Jul 13;15:265. doi: 10.1186/s12913-015-0932-y (PMC4499441; doi:10.1186/s12913-015-0932-y)
Supplement: Additional file 2: — Appendix B. Resident Education Poster. [file 12913_2015_932_MOESM2_ESM.docx]

**Additional file 2: Resident Education Poster**

**Inter-Disciplinary Care Rounds**

***“On Time & On Task”***

Team Schedule Monday -Friday

| **Blue** | **Red** | **White** |
| --- | --- | --- |
| 11:30am | 11:45am | 12:00pm |

1. ***Identify***:
   1. Patient’s name, PCP, and Hospital Day
   2. Main diagnosis or medical issue
   3. Anticipated discharge date
   4. Anticipated discharge disposition
2. ***Summarize:*** goals of care and treatment plan
3. ***Discuss***: interdisciplinary issues in daily care and discharge planning
4. ***Ask***: what was missed and orders to place?

**Working together can make this time**


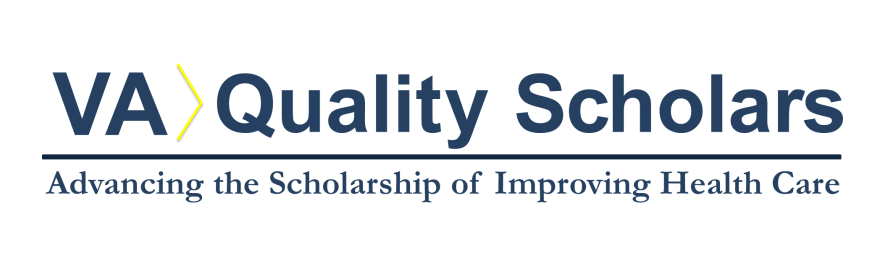
**the most valuable 15 min of your day.**
