# Supplementary figures and images for: Aligning complex processes and electronic health record templates: a quality improvement intervention on inpatient interdisciplinary rounds
Source: BMC Health Serv Res. 2015 Jul 13;15:265. doi: 10.1186/s12913-015-0932-y (PMC4499441; doi:10.1186/s12913-015-0932-y)

**Additional file 3: Electronic Health Record Interdisciplinary Rounds Note Template**


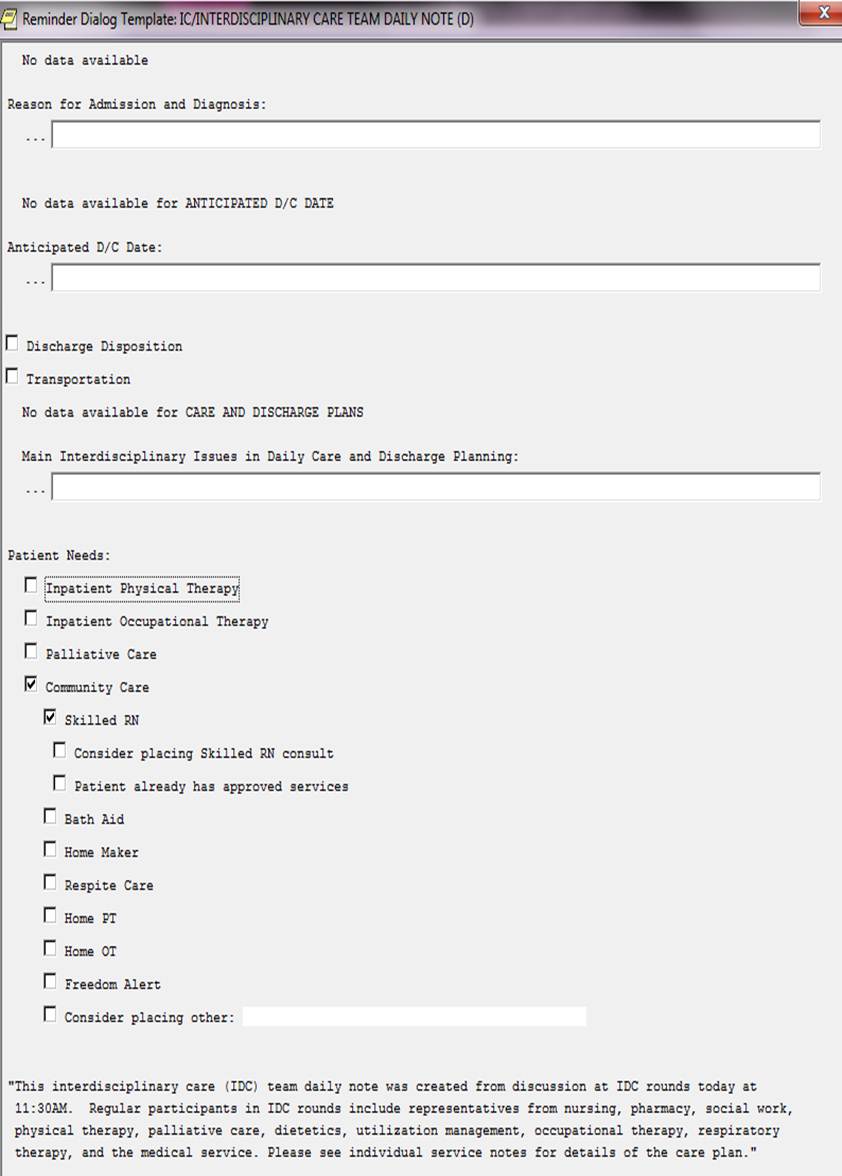

Supplement: Additional file 3: — Appendix C. Electronic health Record Interdisciplinary Rounds Note Template. [file 12913_2015_932_MOESM3_ESM.docx]
