# Supplementary material for: Aligning complex processes and electronic health record templates: a quality improvement intervention on inpatient interdisciplinary rounds
Source: BMC Health Serv Res. 2015 Jul 13;15:265. doi: 10.1186/s12913-015-0932-y (PMC4499441; doi:10.1186/s12913-015-0932-y)
Supplement: Additional file 4: — Appendix D. Pre-Intervention Survey Free-Text Responses. [file 12913_2015_932_MOESM4_ESM.docx]

**Additional file 4: Pre-Intervention Survey Free-Text Responses**

1. *What information do you need from Interdisciplinary Rounds to help you do your job?*

- Medical team's thoughts on discharge time frame.
- Anticipated discharge date, anticipated discharge needs.
- When patients are leaving and how nursing can help facilitate this.
- Discharge date, equipment needs, active problems, changes in patient's status.
- Discharge date, goals to reach for discharge, equipment/supplies needed for discharge
- Clearer expected date of discharge for planning purposes. Sometimes we have little notice and are expected to place immediately.
- When are patients going to be discharged- especially good to know if they will need to be going at a specific time; if they are going to a nursing home, community living center (CLC), etc.; will they need meds at discharge or IV supplies.
- Patients who will be starting tube feedings or total parenteral nutrition (TPN), patients who aren't eating well, patients who would benefit from outpatient (or inpatient) diet education
- What the medicine team is doing (interventions) for the patient that cannot be done on an outpatient basis.

2. *What potential problems are avoided in your line of work by coming to Interdisciplinary Rounds?*

- Improves communication and promotes consistency in plan of care.
- Being prepared for patients starting nutrition support, knowing when patients are going home.
- Learning what all disciplines do-and have done-preparing for discharge.
- Incomplete discharges, making sure patients have all information and are going home with proper services. Also informing teams how patients are the majority of the time on the floor for mental status and physical limits.
- Filling up the inpatient beds as we find out in rounds what patients will be discharging.
- Last minute requests for physical therapy (PT) evaluation. It is helpful to understand the medical team's thought process for the patient stay.
- Last minute referrals for consults, equipment, etc.
- Last minute decisions to discharge can result in things being overlooked, falling through cracks, etc.
- These rounds give us a heads up about how busy the next couple of days will be so that we can staff medication reconciliation appropriately. It also helps us to know if people are going at specific times (ambulance) so we can prioritize their discharge and aren't adding additional charges to the VA by ambulance services for prolonged wait times.

*3. Please list 1-2 ideas on how Interdisciplinary Rounds could be improved to meet your needs.*

- Residents have clearer understanding of team members and their role! Residents need to know about palliative care and refer appropriately.
- Not having so much down time in between teams.
- One physician to report per team (takes less time).
- Quality of information shared and residents being on time.
- Start earlier to identify potential discharges so as not to divert as many patients and teams to come on time every time.
- More timely teams--it seemed to work better when we went from room to room. New teams seem to be unsure/unaware of rounds early each month.
- Better meeting environment. All or more physician team involvement/participation (i.e. all residents)
- More of an estimated guess on d/c day by the teams to aid with our service and weekend coverage; a better understanding by the med teams that the medication reconciliation portion of a discharge takes time to complete. We like to say about one hour, so if a patient is being discharged at a specific time then the discharge note needs to be entered before that pick up time. However, we realize that sometimes this is not always possible; there is a lot of downtime waiting for the teams.
