# Supplementary material for: Aligning complex processes and electronic health record templates: a quality improvement intervention on inpatient interdisciplinary rounds
Source: BMC Health Serv Res. 2015 Jul 13;15:265. doi: 10.1186/s12913-015-0932-y (PMC4499441; doi:10.1186/s12913-015-0932-y)
Supplement: Additional file 5: — Appendix E. Post-Intervention Survey Free-Text Responses. [file 12913_2015_932_MOESM5_ESM.docx]

**Additional file 5: Post-Intervention Survey Free-Text Responses**

1.*What behaviors have Resident’s shown in Interdisciplinary Rounds that maximize the meetings effectiveness?*

| - Staying focused with the end in mind (discharge). - Speak briefly about patient’s needs and if patient has no needs move to the next patient. - Following the (IDR rounds) guide. - Acknowledge the various disciplines (in regard to patients’ needs) vs. just stating the discharge date. Providing a brief summary of patient’s overall medical care and what still needs to be adderessed. - Discussing key points that are relevant to patient's care. - Most have been providing appropriate information and listen to requests/recommendations. - Most seem willing to participate and understand the importance of attending. There are the occasional residents that seems like they would rather be somewhere else. - Open to questions. - Outlining key issues for each patient. Clarifying any factors impacting length of stay. - A collaborative perspective- it’s helpful. - Receptiveness to input from team questions asked. |
| --- |

2. *Please List 1 or 2 ideas on how Interdisciplinary Rounds could be improved to meet your needs.*

| - Less socializing; each discipline stick to their own issues. - Discuss/identify what education might need to be accomplished several days before discharge. This occurs most of the time but I think we need to keep working on it. Place consults early in the admissions as needed vs. day of discharge (e.g., nutrition education). - Focus more on what procedures/workup can be completed as an outpatient. - Less down time between teams. - Have a bigger room. - New to group; may have more input at later time. - Would be nice if provider teams were timely and didn't need to be "found". - Decrease time between team- perhaps a better call; perhaps an intercom verses phone or page might be more effective. |
| --- |
